# The Role of Vericiguat in Heart Failure Therapy: From Clinical Trials to Clinical Practice

**DOI:** 10.31083/RCM39886

**Published:** 2025-08-29

**Authors:** Lucia Tricarico, Michele Correale, Ester Maria Lucia Bevere, Natale Daniele Brunetti, Massimo Iacoviello

**Affiliations:** ^1^Department of Medical and Surgical Sciences, University of Foggia, 71122 Foggia, Italy; ^2^Cardiothoracic Department, Ospedali Riuniti University Hospital, 71122 Foggia, Italy

**Keywords:** heart failure, vericiguat, soluble guanylate cyclase, nitric oxide, worsening heart failure

## Abstract

Heart failure with reduced ejection fraction (HFrEF) is a progressive condition that is associated with high rates of morbidity, frequent hospitalizations, and significant mortality. Despite advancements in guideline-directed medical therapy (GDMT), many patients continue to be at risk for worsening heart failure (WHF). Vericiguat is a novel soluble guanylate cyclase (sGC) stimulator that targets the impaired nitric oxide (NO)–sGC–cyclic guanosine monophosphate (cGMP) pathway. Thus, by improving vascular and myocardial function, vericiguat offers a promising therapeutic option for patients with HFrEF who remain symptomatic despite receiving optimal medical treatment. This review explores the pathophysiological rationale, mechanism of action, and clinical evidence supporting the use of vericiguat. We analyze data from key randomized controlled trials (RCTs), such as SOCRATES-REDUCED and VICTORIA, as well as meta-analyses, to assess the efficacy and safety of using vericiguat in HFrEF. Additionally, we review real-world studies to evaluate the applicability of vericiguat in clinical practice.

## 1. Introduction

Heart failure (HF) is a complex clinical condition which is associated with high levels of illness, frequent hospitalizations, and a high mortality rate HF. Moreover, it significantly impacts patients’ quality of life and places a substantial strain on healthcare systems [1]. HF patients are classified based on the value of their left ventricular ejection fraction (LVEF). HF with reduced ejection fraction (HFrEF), defined as an ejection fraction below 40%, is characterized by pathophysiology strongly related to the activation of neurohumoral pathways, including the sympathetic nervous system, the renin-angiotensin-aldosterone system (RAAS), and vasoactive peptides. These maladaptive responses contribute to disease progression [1, 2, 3]. Current treatment strategies focus on four cornerstone drug classes: β-blockers, angiotensin receptor-neprilysin inhibitors (ARNi), mineralocorticoid receptor antagonists (MRAs), and sodium-glucose cotransporter-2 inhibitors (SGLT2i). While these therapies have demonstrated significant benefits in reducing cardiovascular mortality and HF-related hospitalizations, a residual risk remains, particularly in patients experiencing worsening HF.

Worsening HF (WHF) is a critical condition marked by the deterioration of symptoms in patients with chronic HF despite guideline-directed medical therapy (GDMT). It often necessitates urgent treatment escalation, typically with diuretics and/or hospital readmission [4]. Moreover, after WHF, a high rate of readmission during the vulnerable postdischarge phase (first six months) and a high mortality are observed. Given these challenges, there is a need for additional therapeutic options beyond standard GDMT to improve outcomes in patients who remain symptomatic despite optimal medical therapy.

In this setting, vericiguat, a soluble guanylate cyclase (sGC) stimulator, represents a second-line therapy for patients with HFrEF and WHF despite GDMT. By targeting the nitric oxide (NO)-sGC-cyclic guanosine monophosphate (cGMP) pathway, vericiguat enhances cGMP signalling, resulting in improved myocardial and vascular function. Additionally, HF patients frequently present with multiple comorbidities, such as renal dysfunction, arterial hypotension, and electrolyte disorders, which often prevent the improvement of guideline-directed therapies [5]. Emerging evidence from randomized clinical trials, substudies, and meta-analyses suggests that vericiguat is well tolerated and effective in patients with worsening HF, reducing hospitalizations and mortality.

This review explores the potential role of vericiguat as an emerging treatment in HFrEF, from clinical evidence to real-world practice.

## 2. From NO-sGC-cGMP Pathway to Vericiguat: Mechanism of Action and Systemic Effects

**Fig. 1.**
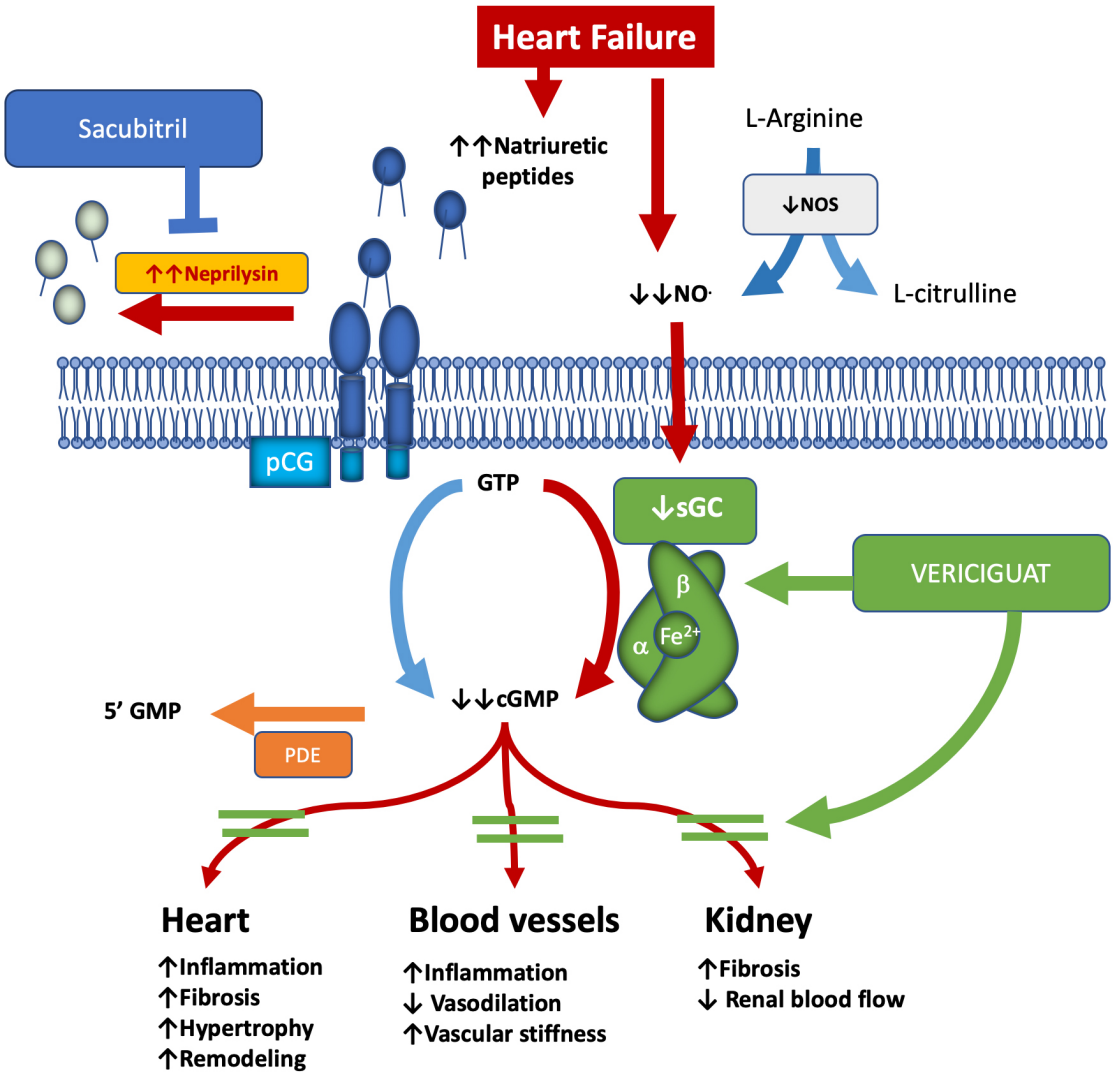
**NO–sGC–cGMP pathway in heart failure and vericiguat: mechanism & effects**. The arrows indicates the effects of the NO-cGC-cGMP pathway on the heart, blood vessels and kidney, i.e., up arrow the increase, down arrow the decrease. cGMP, cyclic guanosine monophosphate; GTP, guanosine triphosphate; GMP, guanosine monophosphate; NO, nitric oxide; NOS, nitric oxide synthase; PDE, phosphodiesterase; pGC, particulate guanylyl cyclase; sGC, soluble guanylyl cyclase.

The progression of HF is closely associated with dysfunction in the NO, sGC, cGMP pathway. This pathway is essential for regulating vascular tone, maintaining endothelial function, and ensuring myocardial integrity. Vericiguat, an oral stimulator of sGC, enhances its activity and stabilizes the nitrosyl-heme complex, leading to an increase in cGMP production. Consequently, vericiguat promotes vasodilation, reduces platelet aggregation, and provides protection against damage to both the myocardium and blood vessels [6, 7] (Fig. 1).

In HF, endothelial dysfunction, inflammation, and oxidative stress reduce the availability of NO. This reduction impairs the synthesis of cGMP and contributes to the progression of the disease. Vericiguat helps restore sGC function, even in low-NO environments. This restoration aids in maintaining vascular homeostasis and provides anti-inflammatory and antifibrotic effects [7].

Elevated cGMP activates protein kinase G (PKG), which inhibits fibroblast proliferation and collagen production, thereby reducing myocardial fibrosis. This process improves diastolic function and decreases cardiomyocyte hypertrophy. Clinical studies have demonstrated that vericiguat reduces both left ventricular end-diastolic and end-systolic volumes, leading to improved left ventricular ejection fraction [8, 9].

Direct data on right ventricular remodeling are limited. However, vericiguat may enhance right ventricular morphology and function by modulating vascular tone, reducing pulmonary artery pressure, and improving cardiac output. Some studies suggest improvements in global ventricular remodeling, including right ventricular parameters, particularly in patients with biventricular dysfunction [10].

Additionally, the regulation of cGMP by natriuretic peptides further enhances the mechanism of vericiguat [11].

Vericiguat has been specifically designed to offer improved metabolic stability, featuring a longer half-life and lower clearance compared to riociguat. This makes it suitable for once-daily dosing with minimal drug interactions. Due to its pharmacological properties and mechanism of action, vericiguat has demonstrated significant cardiovascular effects in preclinical studies. For example, in an isolated rat heart model, it effectively reduced coronary perfusion pressure without affecting heart rate or contractility. Furthermore, research involving animal models of hypertension, heart failure, and kidney disease has highlighted its dose-dependent anti-fibrotic and organ-protective effects, which are consistent with the mode of action of sGC stimulators [7].

These compounds have been shown to limit cardiomyocyte hypertrophy *in vitro* [12], reduce left ventricular and vascular fibrosis [13, 14], decrease infarct size [15], and preserve ejection fraction following myocardial infarction. Additionally, they provide protection against cardiac and renal damage, improving survival rates in hypertensive rats while reducing cardiac hypertrophy and enhancing renal function [16, 17].

While predictive models for HF remain limited, certain animal studies effectively replicate cardiovascular morbidity and characteristics of HF. For instance, renin transgenic rats treated with the NOS inhibitor L-NAME exhibited endothelial dysfunction, nitric oxide depletion, and rapidly progressive hypertension-associated organ injury, leading to elevated morbidity and mortality rates [17]. Furthermore, chronic oral administration of vericiguat significantly reduced mortality and cardiac hypertrophy while lowering plasma atrial natriuretic peptide concentrations. It also prevented severe kidney injury, as evidenced by marked proteinuria.

Given that the NO–sGC–cGMP signaling pathway is disrupted in cardiovascular diseases and heart failure, preclinical evidence strongly supports vericiguat’s ability to restore this pathway, reinforcing its potential as a therapeutic option for these conditions.

Vericiguat shows promising effects in the treatment of pulmonary hypertension (PH), particularly in cases related to heart failure and endothelial dysfunction. Recent studies indicate that vericiguat can induce vasodilation in pulmonary arteries, reduce inflammation, and promote bronchodilation. In *ex vivo* rat lung models, it has been shown to dilate pulmonary arteries and decrease airway resistance, both of which could be beneficial for managing PH. However, when compared to riociguat, another sGC stimulator approved for pulmonary arterial hypertension, vericiguat appears to be less potent in reducing pulmonary artery pressure [18]. In a clinical case involving PH due to left heart disease, vericiguat improved right ventricular–pulmonary artery coupling and reduced pulmonary artery systolic pressure. These findings suggest potential benefits for select patients with combined pre- and post-capillary pulmonary hypertension [19]. It is important to note that vericiguat is not currently approved for treating pulmonary arterial hypertension or chronic thromboembolic pulmonary hypertension (CTEPH), and its use in these conditions is still under investigation. Ongoing trials, such as ARETHA, are examining its effects on diastolic pulmonary arterial pressure in heart failure patients [20].

Additionally, vericiguat may decrease the renal arterial resistance index (RI) by increasing cGMP production, which promotes vasodilation and relaxation of renal vascular smooth muscle cells. This vasodilation reduces renal vascular resistance, leading to a lower resistive index, a parameter calculated using Doppler ultrasound that quantifies resistance in renal arteries. By improving renal blood flow and reducing intrarenal pressure, vericiguat may help preserve kidney function, particularly in patients with heart failure who also have compromised renal perfusion and function [21, 22]. However, direct clinical evidence specifically linking vericiguat to reductions in renal RI is still emerging. A recent article in the International Journal of Cardiology observed changes in RI before and after vericiguat administration, suggesting a potential benefit [23].

## 3. Evidence Medicine (Trials and Meta-analysis)

Several large-scale clinical trials and meta-analyses have evaluated the efficacy and safety of Vericiguat in HF subjects. The SOCRATES-REDUCED (Soluble guanylate Cyclase stimulatoR in heArTfailurE Study) [24, 25] and VICTORIA (vericiguat in patients with Heart Failure and Reduced Ejection Fraction) [26] trials have been the primary studies assessing the efficacy, safety and tolerability of vericiguat in patients with HFrEF (Table 1, Ref. [25, 26, 27]).

**Table 1.** **Key clinical trials**.

Trial	Patient population	Key findings	Inclusion criteria	Exclusion criteria
SOCRATES-REDUCED [25]	HFrEF patients (NYHA II–IV, LVEF <45%) with recent worsening	No significant change in NT-proBNP, but higher doses reduced hospitalization and mortality	- Chronic HF (NYHA II–IV)	- IV inotropes use after admission
			- LVEF <45%	- Concurrent nitrates or PDE5 inhibitors
			- Worsening HF within 4 weeks	- Recent ACS (within 60 days)
			- Elevated BNP/NT-proBNP (NT-proBNP ≥1000 pg/mL or BNP ≥300 pg/mL in sinus rhythm, or NT-proBNP ≥1600 pg/mL or BNP ≥500 pg/mL in atrial fibrillation)	- Listed for transplant/VAD
				- GFR <30 mL/min
VICTORIA [26]	HFrEF patients (NYHA II–IV, LVEF <40%) with recent decompensation	10% reduction in CV death/HF hospitalization; particularly in those with NT-proBNP <8000 pg/mL	- Chronic HF (NYHA II–IV)	- GFR ≤15 mL/min
			- LVEF <40%	
			- Recent worsening HF event	
			- GFR >15 mL/min	
VERITA (real- world) [27]	Real-world HFrEF patients with recent worsening	Improved NYHA class, reduced hospitalizations, and good tolerability	- HFrEF with recent worsening requiring IV therapy	- Systolic BP <100 mmHg at vericiguat initiation
			- On GDMT	
			- Systolic BP ≥100 mmHg	

ACS, Acute coronary syndrome; BP, blood pressure; CV, cardiovascular; GDMT, guideline-directed medical therapy; GRF, Glomerular filtration rate; HF, Heart failure; HFrEF, Heart failure with reduced ejection fraction; IV, intravenous; LVEF, Left ventricular ejection fraction; NT-proBNP, N-terminal pro-brain natriuretic peptide; NYHA, New York Hearth Association; PDE5, phosphodiesterase type 5 inhibitors; VAD, ventricular assist device.

The SOCRATES-REDUCED trial [25] aimed to establish the correct dose of vericiguat for patients with HFrEF. Key inclusion and exclusion criteria are detailed in Table 1. A total of 456 patients with chronic HFrEF who experienced recent worsening heart failure were enrolled. Among these, 92 patients were assigned to the placebo group, while the remaining participants received one of four doses of vericiguat (1.25 mg, 2.5 mg, 5 mg, or 10 mg) for 12 weeks. The primary endpoint was the change in N-terminal pro-brain natriuretic peptide (NT-proBNP) levels from baseline to week 12. The main analysis compared the three highest-dose vericiguat groups to the placebo group, while the secondary analysis evaluated the dose-response relationship between vericiguat and the primary endpoint. Although the primary analysis did not show a statistically significant difference between the vericiguat and placebo groups (*p* = 0.15), higher doses of vericiguat were associated with a significant reduction in NT-proBNP levels (*p* < 0.02). Furthermore, there was a notable decrease in mortality and hospitalization rates, particularly among patients receiving the two highest doses of vericiguat [24, 25].

The VICTORIA trial [26] is a randomized, phase III, double-blind study that included 5050 patients with chronic HFrEF (New York Hearth Association (NYHA) class II–IV) with a glomerular filtration rate (GFR) above 15 mL/min. Participants were initially randomized to receive 2.5 mg of vericiguat or a matching placebo. Their doses were then escalated—first to 5 mg and eventually to a target dose of 10 mg once daily—in a blinded manner, based on assessments of blood pressure and clinical symptoms. The primary endpoint was defined as the composite of cardiovascular death or the first hospitalization for heart failure. Results showed a 10% reduction (HR 0.9) in the composite endpoint among patients treated with vericiguat. This reduction was primarily due to a decrease in HF hospitalizations rather than a decrease in mortality. The greatest benefit was observed in patients with NT-proBNP levels below 8000 pg/mL, particularly when BNP was less than 4000 pg/mL (HR 0.90; 95% CI, 0.82 to 0.99). In contrast, patients with NT-proBNP levels above 8000 pg/mL did not show a significant difference in outcomes compared to the placebo group. While vericiguat did not adversely affect the GFR, its impact on patients with a GFR below 15 mL/min was not examined [28].

These studies recruited patients with more severe forms of HFrEF compared to other trials. For instance, the proportions of patients classified as NYHA class III–IV were higher in the SOCRATES-REDUCED (47%) and VICTORIA (41%) trials than in the PARADIGM-HF [28] and DAPA-HF [29] studies (25% and 32%). Baseline average NT-proBNP levels were also higher in SOCRATES-REDUCED (3076 pg/mL) and VICTORIA (2861 pg/mL) compared with PARADIGM-HF (1608 pg/mL) and DAPA-HF (1347 pg/mL). Furthermore, a larger percentage of patients in the VICTORIA study had implantable devices (42%) compared to those in PARADIGM (21%) or DAPA-HF (33%). Regarding comorbidities, the VICTORIA study reported a higher incidence of diabetes (46.9% vs. 34.6% in PARADIGM and 41.8% in DAPA-HF) and a higher stroke rate (11.5% vs. 8.5% in PARADIGM). Additionally, the COMMANDER HF trial [30] focused exclusively on patients with ischemic heart failure, while VICTORIA included a more heterogeneous patient population.

The VICTOR (Vericiguat Global Study in Participants with Chronic Heart Failure) trial is currently ongoing and aims to establish the efficacy and safety of vericiguat in patients with an ejection fraction of ≤40% who have not recently experienced worsening heart failure, while they continue to receive guideline-directed HFrEF therapy [31]. Additional meta-analyses have reinforced the findings of this trial, highlighting the role of vericiguat in optimizing heart failure management, particularly in high-risk populations who experience persistent symptoms despite GDMT.

A network meta-analysis (NMA) of phase 3 trials, including VICTORIA and PARADIGM-HF, evaluated the efficacy of vericiguat compared to sacubitril/valsartan in treating HFrEF. The results showed no significant difference (hazard ratio [HR]: 0.88, 95% confidence interval [CI]: 0.62–1.23), confirming vericiguat’s non-inferiority within the 1.24 margin. Sensitivity analyses further supported these findings, indicating that vericiguat is a viable treatment option [32].

Another NMA compared four HFrEF treatments: vericiguat, sacubitril/valsartan, SGLT2 inhibitors (SGLT2i), and standard care, using data from six trials [33]. The results showed that SGLT2i led to the greatest reduction in heart failure hospitalizations, but it did not significantly affect cardiovascular mortality when compared to vericiguat (HR 0.88, 95% CI: 0.63–1.22) or sacubitril/valsartan (HR 1.04, 95% CI: 0.88–1.24). Based on Surface Under the Cumulative Ranking curve (SUCRA) scores, vericiguat ranked third in overall efficacy.

Recent evidence has highlighted the effectiveness of therapies for HFrEF that do not modulate the renin-angiotensin-aldosterone system, including vericiguat, SGLT2i, and omecamtiv mecarbil. A network meta-analysis of 12 randomized controlled trials involving 23,861 patients assessed these treatments, revealing that both SGLT2 inhibitors and vericiguat were more effective than placebo in reducing the primary composite endpoint of heart failure hospitalization or cardiovascular death (HF-CVD). In contrast, omecamtiv mecarbil did not demonstrate significant benefits [34].

SGLT2i significantly reduced the risk of cardiovascular disease-related heart failure (CVD-HF) compared to placebo, vericiguat, and omecamtiv mecarbil, with relative risks (RR) of 0.77, 0.84, and 0.80, respectively. There was no significant difference between vericiguat and omecamtiv mecarbil, with an RR of 0.95 [34]. SGLT2i also outperformed placebo and omecamtiv mecarbil across all secondary endpoints, including cardiovascular death, all-cause mortality, and HF hospitalization. Furthermore, SGLT2i showed superiority over vericiguat in reducing HF hospitalizations. Overall, SGLT2i emerged as the most effective therapy, followed by vericiguat, omecamtiv mecarbil, and placebo.

The safety of vericiguat in patients with coronary artery disease was evaluated in a meta-analysis of three randomized controlled trials (RCTs) [35]. The results indicated a minor, clinically insignificant reduction in systolic blood pressure (1.4–10 mmHg) and diastolic blood pressure (0.4–6 mmHg), along with a slight increase in heart rate (1.8–7 bpm). Although there was no significant difference in severe adverse events between vericiguat and placebo (odds ratio [OR] = 1.97, 95% confidence interval [CI] = 0.39–9.91, *p* = 0.41), vericiguat was associated with a notably higher overall rate of adverse events (OR = 4.04, 95% CI = 2.17–7.52, *p* < 0.001). The study suggests that vericiguat is generally safe, but further clinical trials are needed for confirmation.

Substudies of the VICTORIA trial produced both intriguing and controversial results. One analysis showed that vericiguat does not significantly impact renal function compared to placebo [36], indicating that its effects are independent of baseline estimated glomerular filtration rate (eGFR) and worsening renal function. Additionally, no increased risk of developing atrial fibrillation (AF) was observed in patients treated with vericiguat, regardless of the presence of AF at baseline [37].

Another substudy investigated whether vericiguat reduced HF hospitalizations compared to placebo [38]. The findings revealed no meaningful difference in hospitalization or cardiovascular death rates between the two groups (unadjusted HR, 0.89 [95% CI, 0.81–0.97]; adjusted HR, 0.92 [95% CI, 0.84–1.01]). However, an NT-proBNP threshold of 2816 pg/mL was identified, below which vericiguat appeared to provide clinical benefits in reducing re-hospitalizations and all-cause mortality. Despite these findings, concerns about vericiguat’s overall effectiveness remain, as post-hospitalization mortality was high in both groups (48.6% for vericiguat vs. 44.1% for placebo).

The safety and tolerability of vericiguat have been evaluated in high-risk patients, including those over 75 years old, those with low baseline systolic blood pressure (SBP), and patients already receiving ARNI therapy [39]. In this subgroup, the incidence of symptomatic hypotension or syncope was found to be comparable to that of the placebo group, and the efficacy of vericiguat remained consistent regardless of initial SBP levels. Although a slight initial decrease in SBP was observed across all groups, including the placebo, continued treatment resulted in stabilization.

One reported side effect of vericiguat is a modest decline in hemoglobin levels. A post hoc analysis indicated that, at 96 weeks, the mean difference in hemoglobin levels between the vericiguat and placebo groups was 0.255 g/dL [40]. However, the hemoglobin/hematocrit ratio remained stable throughout the trial, and this decline in hemoglobin was not associated with vericiguat’s clinical benefits in reducing heart failure hospitalization or cardiovascular death. Therefore, issues related to anemia or hemoglobin reduction should not affect treatment decisions for patients with HFrEF.

## 4. Real-Life and Real-World Studies

An important consideration for new clinical trial evidence is how applicable the results are to real-world patients, who often differ significantly from trial participants. Typically, real-world patients are older, have poorer renal function, more comorbidities, and greater overall frailty.

An analysis of the PINNACLE registry, which includes over 14,000 patients with HFrEF, identified 3754 patients (26%) who met eligibility criteria similar to those used in the VICTORIA trial [41]. These patients had characteristics comparable to the VICTORIA placebo group but exhibited a higher annual hospitalization rate (35.8% vs. 29.6%). This suggests that at least one in four real-world patients could benefit from vericiguat. Similarly, a study from a Korean registry found that 58% of 5625 hospitalized heart failure patients met the VICTORIA eligibility criteria. These findings indicate that a significant proportion of real-world patients with HFrEF may be suitable for vericiguat treatment.

The VERITA study evaluated the clinical profile, safety, and outcomes of vericiguat in a real-world cohort of HFrEF patients who had experienced recent worsening episodes [27]. Among the 103 patients initially included, 52 had at least six months of follow-up. The mean age of participants was 71.3 years, 27.2% were women, and most were receiving guideline-directed therapy. During follow-up, there was a significant improvement in the NYHA functional class (*p* < 0.001), and quality-of-life scores (EQ-5D and visual analogue scale (VAS)) increased (*p* = 0.032 and *p* = 0.005, respectively). Vericiguat was well tolerated, with 13.5% of patients experiencing symptomatic hypotension and 11.5% discontinuing treatment. Most patients (78.8%) achieved the target dose of 10 mg. Heart failure-related hospitalizations decreased from an average of 2.3 to 0.79 per year (*p* < 0.001), and the overall mortality rate was 7.7%, with half of the deaths attributed to heart failure. The study concluded that vericiguat is associated with reduced hospitalizations, improved functional status, and a favorable safety profile in a real-world setting.

Additionally, a real-world study involving 73 HFrEF patients found that vericiguat significantly improved left ventricular reverse remodeling. The treatment led to reductions in end-diastolic and end-systolic volumes while increasing ejection fraction (*p* < 0.001) [8]. These benefits were observed in all patients, including those who could not receive quadruple medical therapy. The incidence of cardiovascular events did not differ significantly between groups (log-rank *p* = 0.555), indicating that vericiguat is effective regardless of whether patients are on guideline-directed therapy.

The effects of Vericiguat on right ventricular function were assessed in a real-world setting. Hashimoto *et al*. [42] investigated its efficacy in patients with HFrEF, particularly focusing on the influence of Vericiguat on right ventricular (RV) to pulmonary artery (PA) uncoupling and left ventricular remodeling. A retrospective analysis of 63 patients revealed significant reductions in plasma BNP levels and improvements in left ventricular function, as seen through decreased end-diastolic and end-systolic volumes. Additionally, there was enhanced RV-PA coupling, indicated by an increased tricuspid annular plane systolic excursion (TAPSE) to pulmonary artery systolic pressure (PASP) ratio. Importantly, these benefits were observed independently of standard quadruple therapy or episodes of worsening heart failure. While prior research mainly concentrated on left ventricular effects, this study highlights Vericiguat’s potential in treating right ventricular dysfunction, an area with limited established therapies. The findings suggest that early administration in HFrEF patients could help prevent further deterioration by improving overall biventricular function [42].

Another aspect analyzed was the hemodynamic effects of Vericiguat. A small study involving 12 HFrEF patients who experienced worsening heart failure despite receiving guideline-directed therapies showed that a single 2.5 mg dose significantly reduced mean pulmonary artery pressure (MPAP) and pulmonary artery wedge pressure (PAWP) within 30 minutes during right heart catheterization. Furthermore, long-term treatment over 105 days resulted in a sustained reduction in PAWP [43]. Unlike other medications, such as Riociguat, Vericiguat did not significantly impact cardiac index (CI), systemic vascular resistance (SVR), or pulmonary vascular resistance (PVR). These findings suggest that Vericiguat is well tolerated and may enhance cardiac function by lowering left ventricular filling pressures without compromising systemic circulation.

Vericiguat may also positively affect renal function. Research has shown that it significantly reduces the renal arterial resistance index (RRI) at both 30 and 60 days, without affecting eGFR [23]. The RRI is considered an early marker of cardiovascular and kidney dysfunction, and its reduction may indicate a better prognosis. The study suggests that Vericiguat could provide additional benefits beyond heart failure treatment, potentially establishing itself as the “fifth” key therapy alongside standard heart failure medications. Further studies are needed to confirm its long-term effects.

Observational studies and registry analyses indicate that Vericiguat is well-tolerated and provides benefits to various heart failure patients, including the elderly and those with comorbidities or previous hospitalizations. Real-world evidence supports its integration into standard management strategies. A study of 829 Japanese patients who started Vericiguat within a year of its approval found that most had underlying conditions, including hypertension (91.7%), coronary artery disease (71.3%), and diabetes (60.1%) [44]. Within 90 days, over 65% of patients were uptitrated, and 32.3% reached the maximum dose within a median of 34 days. Factors such as outpatient initiation and previous use of ARNI were linked to higher rates of uptitration, whereas age, chronic kidney disease, and anemia did not seem to impact this process. These findings reinforce Vericiguat’s role in heart failure treatment.

However, challenges may arise during the uptitration of the drug. A separate real-world study analyzed 2916 patients on Vericiguat in Germany, with a mean age of 73 years and 28% being women [45]. Only 36% of participants reached the target dose of 10 mg, with slower uptitration observed in women and older patients. Despite this, adherence to the medication was high at 87%, and 67% of patients continued treatment for a year. The use of Vericiguat increased the number of patients receiving quadruple guideline-directed therapy from 29% to 44%. This study highlights strong adherence but also points out issues with dose optimization, particularly among women and elderly patients.

## 5. Clinical Practice

The 2021 ESC guidelines recommend considering vericiguat for patients with HFrEF in NYHA classes II–IV who experience worsening heart failure despite optimal treatment with a beta-blocker, renin-angiotensin system antagonist, and aldosterone antagonist (Class IIb, Level B) [1]. Similarly, the ACC/AHA/HFSA guidelines suggest its use in high-risk patients who are already on GDMT, which may include SGLT2 inhibitors. However, there is currently no data to confirm additional benefits of combining vericiguat with SGLT2 inhibitors [46].

The risk of rehospitalization should ideally be aligned with the criteria from the VICTORIA trial [28]. This includes elevated natriuretic peptide levels, specifically BNP ≥300 ng/L or NT-proBNP ≥1000 ng/L in sinus rhythm, and BNP ≥500 ng/L or NT-proBNP ≥1600 ng/L in atrial fibrillation. However, the efficacy of vericiguat is reduced in patients with very high baseline NT-proBNP levels, particularly those above 8000 pg/mL, as observed in the subgroup analysis of the VICTORIA trial. This highlights the limited benefit of vericiguat in end-stage or severely decompensated patients, emphasizing the need for careful patient selection [26, 47]. Additional criteria for considering vericiguat include a heart failure-related hospitalization within the past six months or the administration of intravenous diuretics within the past three months. Before initiating treatment, it is essential to confirm clinical stabilization, particularly concerning volume status and blood pressure, as vericiguat should not be started in patients with symptomatic hypotension or a systolic blood pressure of less than 100 mmHg. The recommended starting dose is 2.5 mg per day, which can be titrated to 10 mg per day based on patient tolerance.

Once treatment begins, dose titration is guided by SBP. If SBP is ≥100 mmHg and the patient is not yet on the 10 mg target dose, the dose should be increased. If SBP is ≥100 mmHg and the patient is already on 10 mg, or if SBP is between 90 and <100 mmHg, the dose should be maintained. In patients with SBP <90 mmHg who are asymptomatic, the dose should be decreased if currently on 5 or 10 mg, and interrupted if on 2.5 mg. If SBP is <90 mmHg with symptoms, the dose should be interrupted regardless of the dose level. This approach helps ensure the efficacy and safety of vericiguat use in HF management.

As food enhances absorption, it should be taken with meals. Peak plasma concentrations are typically reached within 1–4 hours, and the drug exhibits dose-proportional pharmacokinetics across the therapeutic dose range [26].

Vericiguat has a terminal half-life of approximately 20–30 hours, which supports once-daily dosing. It is highly protein-bound (~98%) and is metabolized primarily by glucuronidation via UGT1A9 and UGT1A1, with a minor role for cytochrome P450 enzymes. The drug is eliminated mainly via renal (53%) and fecal (45%) routes, primarily as metabolites [48]. Dose adjustments are not needed in elderly patients or those with mild-to-moderate renal (eGFR >15 mL/min/1.73 m^2^) or hepatic impairment, but vericiguat is not recommended in severe hepatic failure. It does not require routine electrolyte monitoring, as hyperkalemia was not an exclusion criterion in the VICTORIA study, and treatment with vericiguat was not associated with significant changes in potassium levels [36]. It is contraindicated with other sGC stimulators like riociguat.

**Fig. 2.**
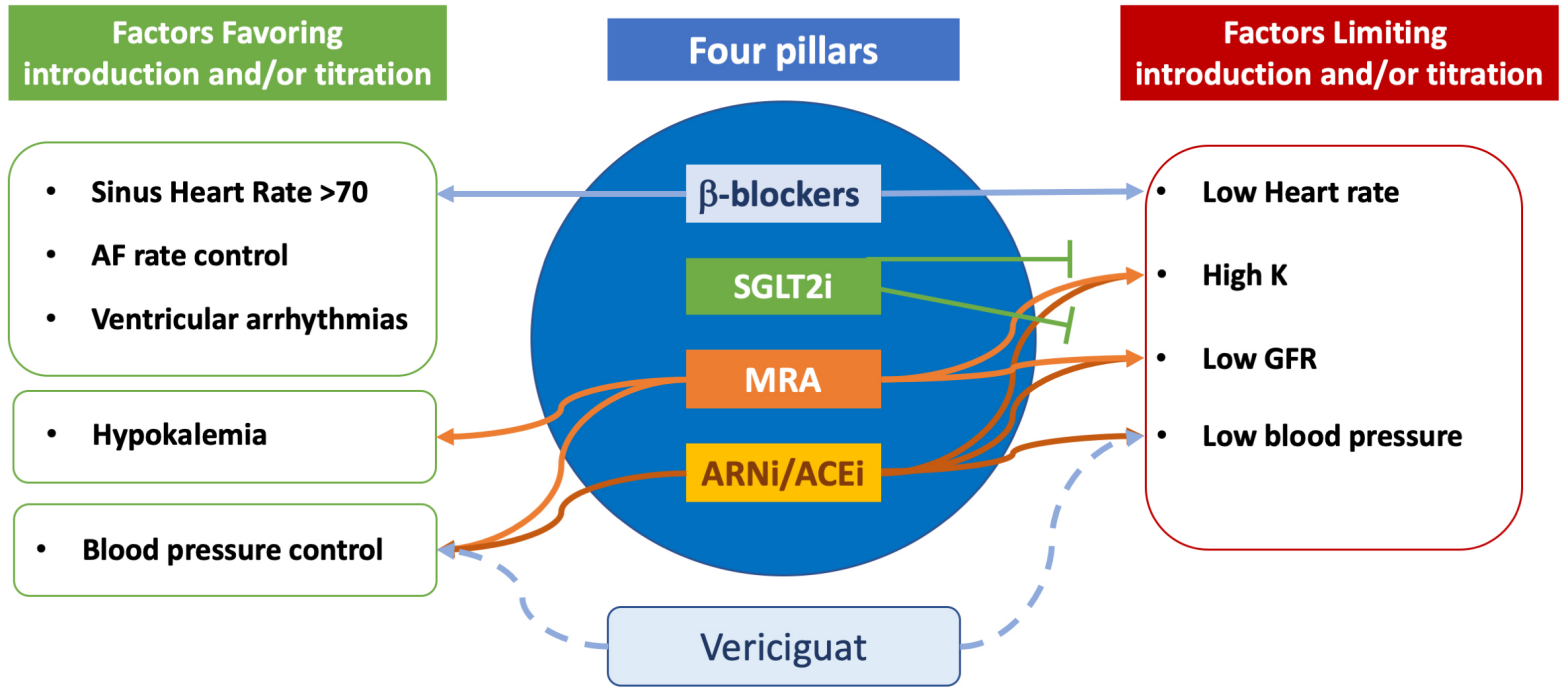
**Incorporating vericiguat in heart failure titration strategy**. In comparison with the four classes currently recommended for treatment of heart failure with reduced ejection fraction, vericiguat presents less factors limiting its use. See the text for more details. ACEi, Angiotensin-Converting Enzyme inhibitor; AF, Atrial fibrillation; ARNi, angiotensin receptor neprylisin inhibitors; GFR, Glomerular filtration rate; K, Potassium; MRA, mineralocorticoid receptor antagonists; SGLT2i, SGLT2 inhibitors.

Vericiguat holds a crucial role in the management of HF patients who experience limitations in titrating foundational therapies. As illustrated in Fig. 2, multiple barriers such as low heart rate, hypotension, impaired renal function, and elevated potassium levels often interfere with the safe up-titration of β-blockers, ACEi/ARNi, and MRAs. Positioned independently of these constraints, vericiguat provides an opportunity to improve clinical stability, without significantly affecting heart rate, kidney function, or potassium balance. Its integration may help create a therapeutic window, allowing better tolerance and optimization of guideline-directed medical therapy.

In selected high-risk heart failure patients, some researchers have begun evaluating the early administration of vericiguat before fully implementing quadruple therapy. A recent review suggests that vericiguat can be considered as an adjunct treatment for patients experiencing worsening heart failure despite being on partial or incomplete GDMT. This is particularly true for patients whose full up-titration of quadruple therapy is limited by intolerance, hypotension, or renal dysfunction. In these cases, vericiguat’s unique mechanism may provide a complementary pathway that helps stabilize patients in the earlier phases of heart failure. Real-world studies indicate that vericiguat can be safely added to other standardized therapies, including ARNI, beta-blockers, mineralocorticoid receptor antagonists (MRA), and SGLT2 inhibitors, leading to improvements in quality of life and reductions in NT-proBNP levels [49]. Preliminary evidence suggests there may be synergistic effects when vericiguat is used with ARNIs (e.g., sacubitril/valsartan), as both therapies target complementary neurohormonal and vasodilatory pathways. Specifically, neprilysin inhibition enhances natriuretic peptides, while sGC stimulation increases cyclic GMP via nitric oxide signaling [50].

Despite this, the consensus remains that quadruple therapy should be the first-line treatment due to its proven mortality benefits. Vericiguat is generally regarded as a second-line or adjunctive option, particularly for patients with persistent symptoms or those who have recently decompensated. When it proves challenging to up-titrate vericiguat to the target dose of 10 mg, practical strategies, such as starting at 5 mg instead of 2.5 mg, can be beneficial. A large observational study found that patients who began taking vericiguat at 5 mg per day were three times more likely to reach the 10 mg target dose compared to those who started at 2.5 mg. This may be due to the simplification of up-titration, which helps reduce patient inertia. The VELOCITY study demonstrated that initiating vericiguat directly at 5 mg per day was well tolerated in over 90% of patients, even among those with recent worsening heart failure, supporting a more confident approach to starting at a higher dose and titrating faster [51].

It is important to monitor for hypotension without overestimating the associated risks. Factors such as older age, chronic kidney disease, anemia, or a history of hypotension were not strongly linked to difficulties in up-titration according to real-world data. Vericiguat is generally well tolerated; in fact, in the Victoria trial, 90% of patients reached the maintenance dose, indicating excellent tolerability [52].

Another critical aspect is enhancing treatment adherence and persistence. Real-world data show that while 70% of patients are titrated beyond 2.5 mg, only 36% achieve the target dose of 10 mg. Therefore, it is essential to increase patient awareness regarding the importance of treatment adherence [45]. Standardizing protocols and educating patients could help prevent clinical inertia and inconsistent follow-up, which may inadvertently contribute to under-titration.

In clinical practice, vericiguat is usually started after a patient has been discharged from the hospital. However, emerging evidence shows that about 10% of patients begin vericiguat therapy during their hospital stay [45]. This early initiation of treatment has significant clinical implications, as it allows for timely optimization of heart failure management during a high-risk period following decompensation, which may help reduce early rehospitalization rates. Starting therapy before discharge also promotes better adherence, as treatment is initiated under clinical supervision. Studies indicate that vericiguat is safe and well-tolerated in hemodynamically stable inpatients, supporting its use in the hospital setting. Nevertheless, its selective application highlights the importance of careful patient assessment, particularly concerning blood pressure, renal function, and overall clinical stability.

## 6. Conclusions

Vericiguat is a valuable addition to HF therapy, specifically targeting the NO-sGC-cGMP pathway to enhance outcomes for patients with worsening HFrEF. Strong evidence from clinical trials, along with emerging real-world data, supports its effectiveness in reducing hospitalizations related to heart failure and lowering cardiovascular mortality. As the management of heart failure continues to advance, incorporating Vericiguat into comprehensive treatment plans will improve patient care and enhance long-term outcomes. Future research should focus on identifying the best patient profiles for its use, optimizing combination therapies, and expanding our understanding of Vericiguat’s broader impact on the pathophysiology of heart failure.
